# Intracranial Cysts: A Single-Institution Experience With 27 Surgically Managed Cases

**DOI:** 10.7759/cureus.64606

**Published:** 2024-07-15

**Authors:** Abdulaziz M Alghamdi, Abdulkarim M Alghamdi, Abdulaziz Hamzah, Abdulrahman H Alsahafi, Reem Adas, Alaa Samkari, Ahmed Lary

**Affiliations:** 1 College of Medicine, King Saud Bin Abdulaziz University for Health Sciences, Jeddah, SAU; 2 Research Office, King Abdullah International Medical Research Center, Jeddah, SAU; 3 Department of Radiology, King Abdulaziz Medical City, National Guard Health Affairs, Jeddah, SAU; 4 Department of Pathology and Laboratory Medicine, King Abdulaziz Medical City, National Guard Health Affairs, Jeddah, SAU; 5 Department of Neurosurgery, King Abdulaziz Medical City, National Guard Health Affairs, Jeddah, SAU

**Keywords:** treatment outcomes, surgical management, radiological evaluation, benign cysts, intracranial cysts

## Abstract

Introduction

Intracranial cysts (ICs) are rare pathologies that are often found incidentally during radiological examinations. They may appear in various brain regions and are categorized as normal, congenital, traumatic, or tumor-associated variants. ICs can be asymptomatic or cause symptoms, such as headaches, visual impairments, or seizures, depending on their size and location. Severe complications include obstructive hydrocephalus, loss of consciousness, and intracranial bleeding. Surgical excision is the most accepted type of management in most ICs.

Objectives

This study aimed to evaluate 27 surgically managed ICs in a tertiary hospital focusing on their clinical, radiological, histopathological, surgical outcomes, and prognosis to enhance understanding and management of these rare, benign cysts.

Methodology

This retrospective cohort study included 27 surgically managed ICs with pathological confirmation in King Abdulaziz Medical City, National Guard Health Affairs, Jeddah, Saudi Arabia, from May 2016 to May 2023. All extracranial and nonsurgically managed cysts have been excluded from this study. Data on demographics, clinical presentations, radiological features, surgical outcomes, and follow-up were retrospectively extracted and analyzed. MRI and CT scans were reviewed to determine cyst characteristics. Surgical outcomes and postoperative complications were recorded. Data were collected via Google Forms and analyzed using the JMP Pro software. Ethical approval was obtained from King Abdullah International Medical Research Center, Jeddah, Saudi Arabia.

Results

The study included 27 ICs: 11 (40.74%) colloid cysts, six (22.22%) epidermoid cysts, five (18.51%) adamantinomatous craniopharyngiomas, two (7.40%) neuroepithelial cysts, and one each of Rathke’s cleft cyst (3.70%), xanthogranuloma (3.70%), and dermoid cyst (3.70%). All 27 cases were surgically managed (100.00%), with gross total resection achieved in 14 (51.85%) cases. Only 12 cases (44.44%) did not develop any surgical complications. Twenty-two cases (81.48%) experienced an improvement in the preoperative presenting symptoms. During the follow-up, only three cases (11.11%) had evidence of recurrence.

Conclusion

This study analyzed 27 ICs of various histopathological types. Each type showed distinct clinical and radiological features. Surgical management generally improved preoperative symptoms with low mortality and recurrence rates, although complications were common. Identifying specific radiological features is crucial for an accurate preoperative diagnosis and optimal surgical outcomes.

## Introduction

Intracranial cysts (ICs) are infrequent pathological occurrences that are typically detected incidentally during radiological evaluations. These cysts can manifest in diverse locations within the brain, such as the ventricles, middle cranial fossa, sellar/suprasellar glands, and pineal glands. They are characterized into various types, including normal variants, congenital, traumatic, and tumor-associated cysts [1,2]. Most ICs are asymptomatic; however, when symptomatic, they can induce a range of clinical manifestations, contingent on their size and location [2]. These symptoms may include headaches, visual impairment, and seizures [3]. Nevertheless, certain cysts can present with severe complications, such as obstructive hydrocephalus, loss of consciousness, intracranial bleeding, and auditory dysfunction [4].

Despite the growing interest in ICs, there is still a dearth of studies that have comprehensively evaluated them using a substantial sample size. Accordingly, this study aimed to describe the clinical, radiological, histopathological, and surgical outcomes of 27 surgically managed ICs treated at our tertiary care center, King Abdulaziz Medical City, Jeddah. By reporting these findings and conducting a detailed analysis, this study can contribute to the existing literature on ICs and offer valuable insights into their identification and management in an evidence-based manner.

## Materials and methods

This retrospective cohort study aimed to describe the cases of surgically managed ICs and explore their clinical presentations, radiological features, surgical outcomes, complications, follow-up, and prognosis. A list of all patients who had ICs from May 2016 to May 2023 was extracted from the neurosurgery department of the National Guard Health Affairs, Jeddah, Saudi Arabia. All the extracranial cysts, nonsurgically managed cysts, or cysts without pathological confirmation were excluded from this study. Utilizing a consecutive sampling technique, the list was filtered only to include surgically managed ICs, with diagnoses confirmed pathologically. No sample calculation was required.

Data collection

The participants’ data were extracted and analyzed retrospectively from electronic patient files in the hospital. Ethical approval was obtained from King Abdullah International Medical Research Center, Jeddah, Saudi Arabia, under protocol number NRJ23J/151/06. 

The data collection sheet included information on demographics, clinical presentations, radiological features, surgical management, postoperative outcomes, and histopathological diagnoses related to surgically managed ICs. Demographic information, including age at diagnosis and sex, was extracted from patients’ files.

Information on presenting symptoms, such as headaches, visual disturbances, and seizures, was collected along with the duration of these symptoms before the diagnosis. Histopathological diagnoses were confirmed by a senior neuropathologist. Brain computed tomography (CT) and magnetic resonance imaging (MRI) findings and diagnoses were recorded and carefully examined by a senior neuroradiologist to confirm their accuracy. CT scans were reviewed to determine cyst density. MRI scans provided T1- and T2-weighted images to determine cyst intensity. In addition, specific MRI features, such as blooming effects, diffusion-weighted imaging (DWI) restriction, and patterns of enhancement, were documented. The precise location and maximum dimensions of each cyst were described based on MRI scans.

Surgical details included the extent of cyst resection, which was categorized as gross total resection (GTR), subtotal resection (STR), or biopsy. Postoperative complications, adjuvant treatment modalities such as radiotherapy, and outcomes regarding preoperative symptoms were recorded. In addition, the postoperative follow-up period was documented in days.

Statistical analysis

John's Macintosh Project (JMP) Pro software version 15 (IMP Statistical Discovery LLC, Cary, North Carolina, United States) was used for data analyses. In the descriptive analysis, categorical variables were presented as frequencies and percentages, whereas numerical variables were displayed as means, standard deviations, medians, and interquartile ranges. Statistical analysis of associations between clinicopathological presentations and mortality was not possible as the mortality rates in this study were very minimal.

Ethical consideration 

A consent form was not needed since the study utilized chart reviews for data collection. The participants' privacy and confidentiality were assured as no sort of identifiers were collected and presented in this study.

## Results

A total of 27 patients with ICs were included in the study. The pathological entities included 11 colloid cysts (40.74%), six epidermoid cysts (22.22%), five adamantinomatous craniopharyngiomas (18.51%), two neuroepithelial cysts (7.40%), and solitary cases of Rathke’s cleft cyst (3.70%), xanthogranuloma (3.70%), and dermoid cyst (3.70%).

Colloid cysts

Eleven patients were included in the study. The median age of the patients was 40 years (range, 33-53 years), and only four cases were males (36.36%). Nine patients (81.81%) presented with headaches, which were the most frequently reported symptom. This was followed by nausea or vomiting in six (54.54%) patients and ataxia in three (27.27%) patients. Hydrocephalus was present in nine patients (81.81%) (Table 1).

**Table 1:** Descriptive analysis of the clinical presentation of the intracranial cyst cases. n: number, IQR: interquartile range

Cyst type/variable	Colloid cyst (n = 11)	Epidermoid cyst (n = 6)	Adamantinomatous craniopharyngioma (n = 5)	Neuroepithelial cyst (n = 2)	Rathke’s cleft cyst (n = 1)	Xanthogranuloma (n = 1)	Dermoid cyst (n = 1)
Age at diagnosis in years, median (IQR)	40 (33 - 53)	26.5 (11.75 - 41)	35 (23 - 44.5)	21.5 (8 - 35)	13	28	23
Gender, n (%) Male	4 (36.36)	4 (66.66)	4 (80.00)	2 (100.00)	0	0	1 (100.00)
Presenting symptoms, n (%)							
Headache	9 (81.81)	3 (50.00)	4 (80.00)	0	1 (100.00)	1 (100.00)	1 (100.00)
Nausea/vomiting	6 (54.54)	2 (33.33)	1 (20.00)	0	0	1 (100.00)	0
Ataxia	3 (27.27)	3 (50.00)	0	0	0	0	1 (100.00)
Seizure	1 (9.09)	0	0	1 (50.00)	0	0	0
Visual disturbances	1 (9.09)	3 (50.00)	4 (80.00)	1 (50.00)	1 (100.00)	1 (100.00)	0
Focal neurological symptoms	1 (9.09)	5 (83.33)	1 (20.00)	1 (50.00)	0	0	0
Tinnitus/decreased hearing	0	3 (50.00)	0	0	0	0	1 (100.00)
Falls/vertigo	1 (9.09)	1 (16.66)	1 (20.00)	0	1 (100.00)	0	1 (100.00)
Fainting episodes	2 (18.18)	0	1 (20.00)	1 (50.00)	0	0	0
Cognitive symptoms	2 (18.18)	0	1 (20.00)	0	0	0	0
Systemic symptoms including fever, malaise, generalized weakness, or decreased oral intake	2 (18.18)	2 (33.33)	2 (40.00)	0	1 (100.00)	0	0
Hormonal deficiencies	0	0	2 (40.00)	0	1 (100.00)	0	0
Hydrocephalus, n (%)	9 (81.81)	2 (33.33)	3 (60.00)	0	0	0	0
Duration of symptoms in months, median (IQR)	1 (0.5 - 6)	13.5 (0.77 - 72)	6 (3 - 30)	5.5 (2 - 9)	96	2	24

CT scan details were available for six (54.54%). A hyperdense appearance was present in four cases (66.66%), and a hypodense appearance was present in two cases (33.33%). MRI details were available for 10 (90.90%). At T1, six cases were hyperintense (60.60%). In addition, in T2, six cases were hyperintense (60.60%). Other MRI scan features included blooming in four cases (40.00%) and peripheral enhancement in four cases (40.00%). The median lesion volume was 71.5 mm (range, 49.75-114 mm) (Table 2).

**Table 2:** Descriptive analysis of the preoperative radiological features of the intracranial cyst cases. n: number, CT: computed tomography, MRI: magnetic resonance imaging, mm: millimeter

Cyst type/variable	Colloid cyst (n = 11)	Epidermoid cyst (n = 6)	Adamantinomatous craniopharyngioma (n = 5)	Neuroepithelial cyst (n = 2)	Rathke’s cleft cyst (n = 1)	Xanthogranuloma (n = 1)	Dermoid cyst (n = 1)
Location, n (%)							
Supratentorial	11 (100.00)	1/5 (20.00)	5 (100.00)	2 (100.00)	0	1 (100.00)	1 (100.00)
infratentorial	0	4/5 (80.00)	0	0	1 (100.00)	0	0
CT scan, n (%)							
Hypodense	2/6 (33.33)	3/3 (100.00)	1/1 (100.00)	1/1 (100.00)	0	1 (100.00)	1 (100.00)
Hyperdense	4/6 (66.66)	0/3	0/1	0/1	0	0	0
Mixed density	0/6	0/3	1/1 (100.00)	0/1	1 (100.00)	0	0
Calcification	0/6	0/3	0/1	0/1	1 (100.00)	0	1 (100.00)
Solid component	0/6	0/3	1/1 (100.00)	0/1	1 (100.00)	0	0
Enhancement	1/6 (16.66)	0/3	0/1	0/1	1 (100.00)	0	0
T1 in MRI scan, n (%)							
Hypointense	1/10 (10.00)	5/5 (100.00)	1/4 (25.00)	2 (100.00)	0	0	-
Isointense	3/10 (30.00)	0/5	1/4 (25.00)	0	0	0	-
Hyperintense	6/10 (60.00)	0/5	0/4	0	1 (100.00)	1 (100.00)	-
Mixed	0/10	0/5	2/4 (50.00)	0	0	0	-
T2 in MRI scan, n (%)							
Hypointense	1/10 (10.00)	0/5	0/4	0	1 (100.00)	0	-
Isointense	0/10	0/5	0/4	0	0	0	-
Hyperintense	6/10 (60.00)	5/5 (100.00)	2/4 (50.00)	2 (100.00)	0	1 (100.00)	-
Mixed	3/10 (30.00)	0/5	2/4 (50.00)	0	0	0	-
Other MRI scan features, n (%)							
Blooming	4/10 (40.00)	0/5	3/4 (75.00)	0	1 (100.00)	0	-
DWI restriction	1/10 (10.00)	4/5 (80.00)	0/4	0	0	0	-
Enhancement	2/10 (20.00)	0/5	1/4 (25.00)	0	0	0	-
Peripheral enhancement	4/10 (40.00)	1/5 (20.00)	4/4 (100.00)	0	0	0	-
Fluid level/ layered enhancement	3/10 (30.00)	0/5	1/4 (25.00)	0	0	0	-
Solid enhancement	3/10 (30.00)	0/5	4/4 (100.00)	0	1 (100.00)	1 (100.00)	-
Volume in mm, median (IQR)	71.5 (49.75 - 114)	141 (89.5 - 144)	82 (74 – 107.5)	127 (69 – 185)	155	43	80

**Figure 1:**
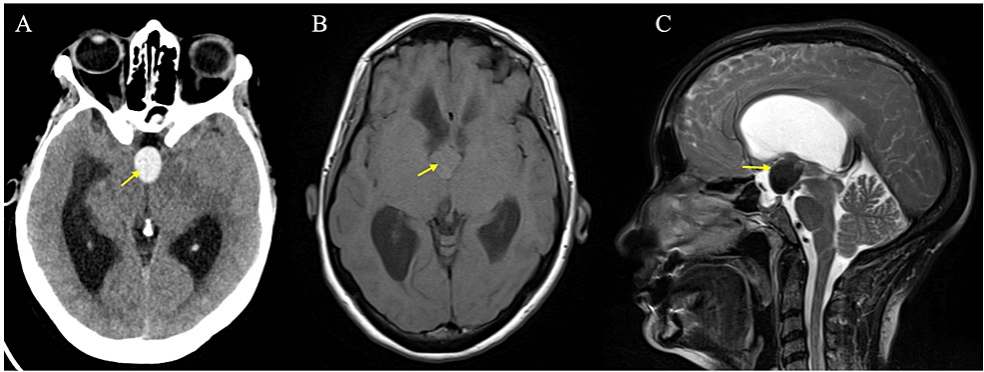
The axial CT scan shows a well-defined hyperdense third ventricular lesion at the level of foramina of Monro (A) (yellow arrow). In an MRI scan, the same lesion is isointense on T1 (B) (yellow arrow) and hypointense on T2 (C) (yellow arrow) with supratentorial hydrocephalous. All the features indicate a diagnosis of a colloid cyst.

**Figure 2:**
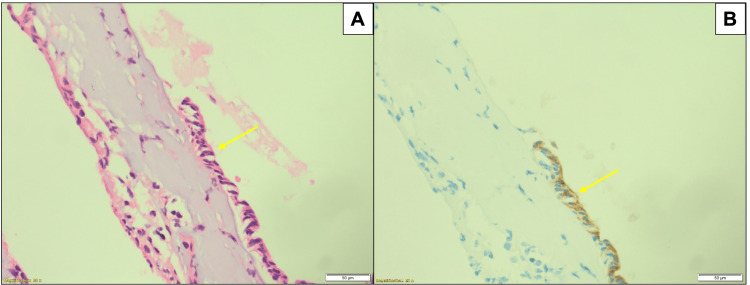
The hematoxylin and eosin-stained section shows a capsule, partially lined by a simple cuboidal to flattened epithelium (A) (50 μm) (yellow arrow), EMA immunohistochemistry is positive in the epithelium (B) (50 μm) (yellow arrow). All the features indicate a diagnosis of a colloid cyst.

Gross total resection was achieved in seven cases (63.63%). Surgical complications included seizures in three patients (27.27%) and decreased Glasgow coma scale in three patients (27.27%). Compared to the preoperative symptoms, nine patients (81.81%) showed improvement. No recurrence was noted among the cases in a median follow-up of 28 (15.2-50.3) months (Table 3). The radiological and pathological features of one of these cases are shown in Figure 1 and Figure 2.

**Table 3:** Descriptive analysis of the surgical management, outcome, and follow-up of the intracranial cyst cases. n: number, GTR: gross total resection, STR: subtotal resection, GCS: Glasgow Coma Scale, EVD: external ventricular device, VP: ventriculoperitoneal, IQR: interquartile range

Cyst type/variable	Colloid cyst (n = 11)	Epidermoid cyst (n = 6)	Adamantinomatous craniopharyngioma (n = 5)	Neuroepithelial cyst (n = 2)	Rathke’s cleft cyst (n = 1)	Xanthogranuloma (n = 1)	Dermoid cyst (n = 1)
Type of resection, n (%)							
GTR	7 (63.63)	3 (50.00)	3 (60.00)	0	0	0	1 (100.00)
STR	4 (36.36)	3 (50.00)	2 (40.00)	2 (100.00)	1 (100.00)	1 (100.00)	0
EVD, n (%)	10 (90.90)	2 (33.33)	0	0	0	0	1 (100.00)
VP shunt, n (%)	5 (45.45)	2 (33.33)	2 (40.00)	0	0	0	1 (100.00)
Surgical complications, n (%)							
Decreased GCS	3 (27.27)	0	0	0	0	0	0
Seizures	3 (27.27)	0	0	0	0	0	1 (100.00)
Temporary focal neurological deficit	2 (18.18)	0	0	0	0	0	0
Permanent focal neurological deficit	1 (9.09)	3 (50.00)	0	0	0	0	0
Hemorrhagic/ischemic infarctions	2 (18.18)	1 (16.66)	0	0	0	0	0
Mortality	1 (9.09)	0	0	0	0	0	0
Infection	0	1 (16.66)	0	0	0	0	0
Hormonal deficiencies	0	0	4 (80.00)	0	0	0	0
Cognitive symptoms	1 (9.09)	0	0	0	0	0	0
None	4 (36.36)	3 (50.00)	1 (20.00)	2 (100.00)	1 (100.00)	1 (100.00)	0
Adjuvant agents, n (%)							
Radiotherapy	0	0	1 (20.00)	0	0	0	0
Radiotherapy and chemotherapy	0	1 (16.66)	0	0	0	0	0
Patients’ status postoperatively, n (%)							
Improved	9 (81.81)	5 (83.33)	3 (60.00)	2 (100.00)	1 (100.00)	1 (100.00)	1 (100.00)
Stayed the same	0	1 (16.66)	2 (40.00)	0	0	0	0
Deteriorated	2 (18.18)	0	0	0	0	0	0
Median follow up period in months, median (IQR)	28 (15.2 – 50.3)	30.1 (8.7 – 49.3)	42.7 (14.63 - 45.58)	4.6 (2.2 - 7)	18	19	16.33
Recurrence, n (%)	0	2 (33.33)	1 (20.00)	0	0	0	0

Epidermoid cysts

Six patients were included in the study. The median age of the patients was 26.5 (11.75-41) years and four cases were males (66.66%). Five patients (83.33) presented focal neurological symptoms (Table 1).

CT scan details were available for three (50.00%). In all three cases, the lesions were hypodense (100.00%). MRI details were available for five (83.33%). At T1, all five cases were hypointense (100.00%). In T2, all five cases were hyperintense (100.00%). Other MRI features included DWI restriction in four cases (80.00%). The median lesion volume was 141 x (89.5-144) mm (Table 2). 

**Figure 3:**
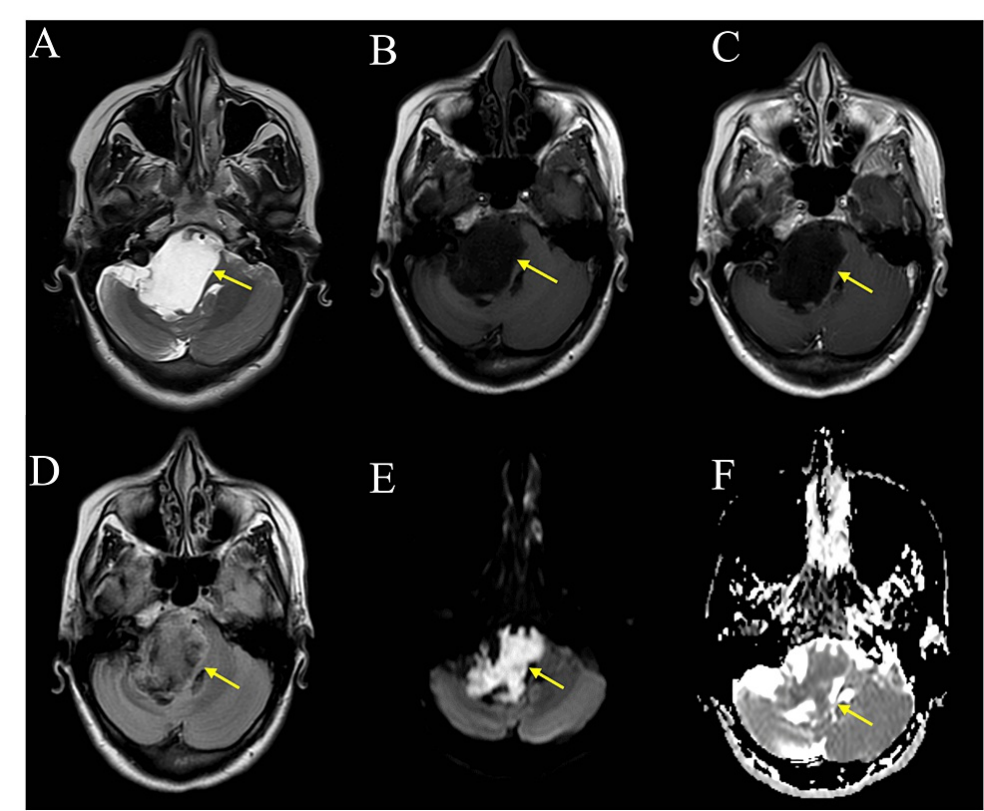
The MRI demonstrates a well-defined lobulated lesion centered in the right cerebellopontine angle cistern. The lesion is heterogeneous and follows cerebrospinal fluid signal intensity on T1 (A) (yellow arrow) and T2 (B) (yellow arrow) images, partially attenuated on FLAIR (C) (yellow arrow), and shows restricted diffusion on DWI (E, F) (yellow arrows). No enhancement is seen on the contrast-enhanced T1 (D) (yellow arrow). All the features indicate a diagnosis of an epidermoid cyst.

**Figure 4:**
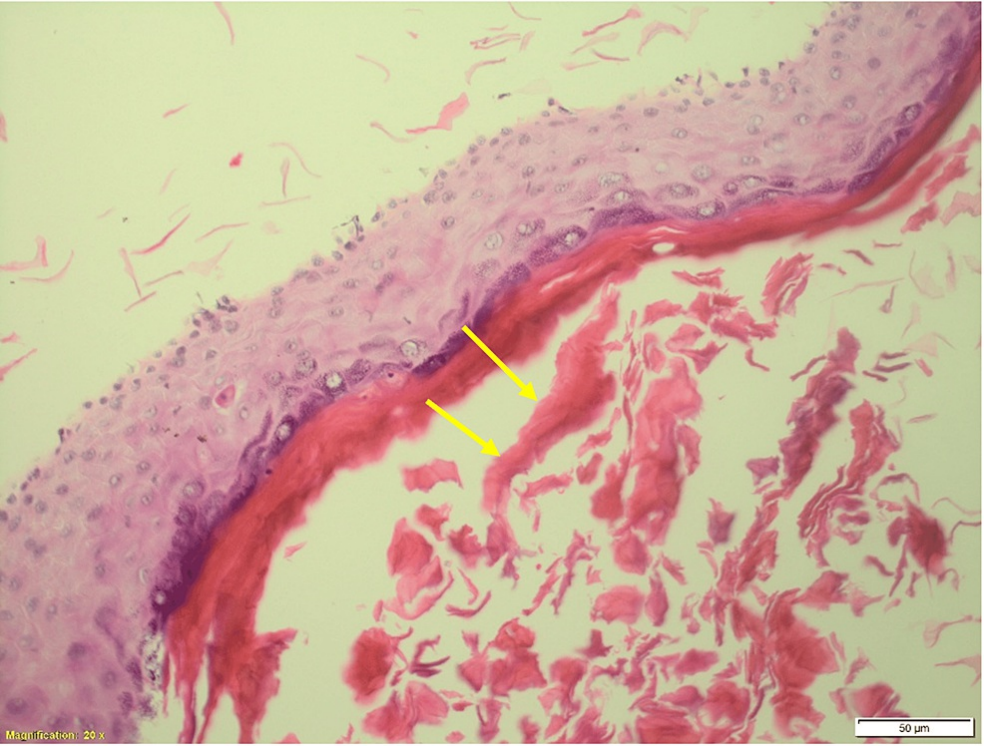
The hematoxylin and eosin-stained section shows a cyst wall lined by stratified squamous epithelium with keratinous contents (yellow arrows) and no adnexal structures (50 μm). All the features indicate a diagnosis of an epidermoid cyst.

GTR was achieved in only three cases (50.00%). Postoperatively, three cases (50.00%) had permanent focal neurological deficits along with many other complications, whereas the other three cases (50.00%) had no surgical complications. Compared with the preoperative symptoms, five patients (83.33%) showed improvement. Two patients had a recurrence in a median of 905.5 (262.5-1481.5) months of follow-up (Table 3). The radiological and pathological features of one of these cases are shown in Figure 3 and Figure 4.

Adamantinomatous craniopharyngiomas

Five patients were included in the study. The median age of the patients was 35 (23-44.5) years, and four cases were male (80.00%). Four patients (80.00%) presented with headaches, and three patients (60.00%) presented with hydrocephalus (Table 1). 

CT scan details were available for one patient (20.00%). In that one case, the lesion was hypodense (100.00%). MRI details were available for four (80.00%). At T1, two patients had mixed intensities (50.00%). At T2, two cases were hyperintense (50.00%), while the other two had mixed intensity (50.00%). Other MRI features included peripheral enhancement and solid enhancement in all four cases (100.00%). The median lesion volume was 82 (74-107.5 mm) (Table 2).

**Figure 5:**
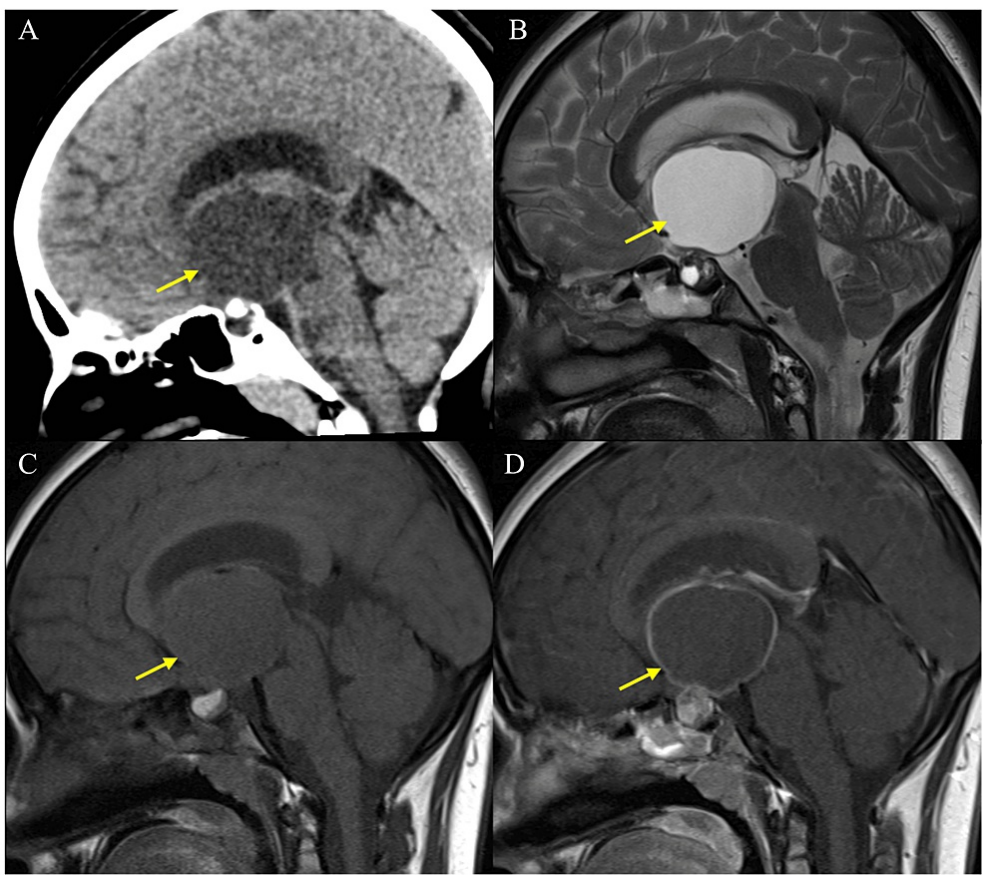
The unenhanced CT of the brain (A) demonstrates a sellar and suprasellar solid and cystic mass with coarse calcification (yellow arrow). The MRI of the sella shows a predominantly high T2 signal intensity cyst (B) (yellow arrow), which is of iso-signal intensity (to grey matter) on T1(C) (yellow arrow). Enhancement of the solid component and the cyst’s wall is noted on the enhanced T1 image (D) (yellow arrow). All the features indicate a diagnosis of an adamantinomatous craniopharyngioma.

**Figure 6:**
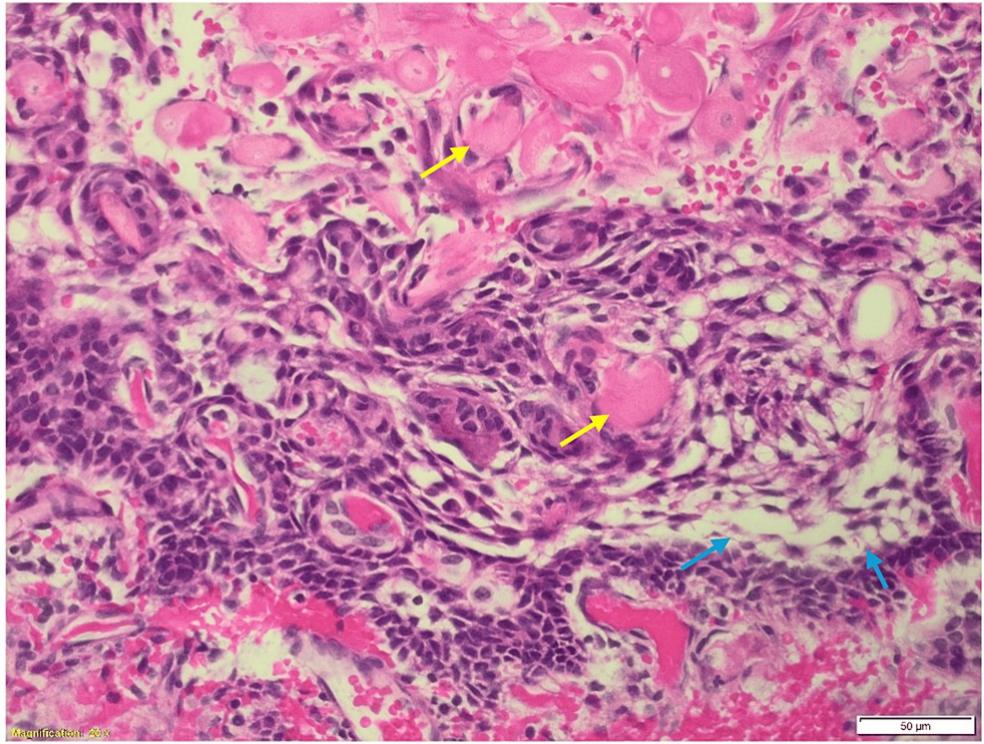
The hematoxylin and eosin-stained section shows distinctive adamantinomatous epithelium, loose “stellate reticulum” (blue arrows), and “wet keratin” (yellow arrows) (50 μm). All the features indicate a diagnosis of an adamantinomatous craniopharyngioma.

GTR was achieved in three cases (60.00%). Four patients (80.00%) developed postoperative hormonal deficiency. Compared with the preoperative symptoms, three patients (60.00%) showed improvement. Only one patient had a recurrence in a median of 42.7 (14.63-45.58) months of follow-up (Table 3). The radiological and pathological features of one of these cases are shown in Figure 5 and Figure 6.

Neuroepithelial cysts

Two patients were included in the study. The median age of the patients was 21.5 (8-35 years), and both were male (100.00%). Preoperative symptoms included seizures (50.00%), visual disturbances (50.00%), focal neurological symptoms (50.00%), or fainting episodes (50.00%) (Table 1). 

CT scan details were available for one patient (50.00%). In that one case, the lesion was hypodense (100.00%). MRI details were available for two (100.00%). At T1, both cases were hypointense (100.00%). At T2, both cases were hyperintense (100.00%), and the median lesion volume was 127 (69-185) mm (Table 2).

**Figure 7:**
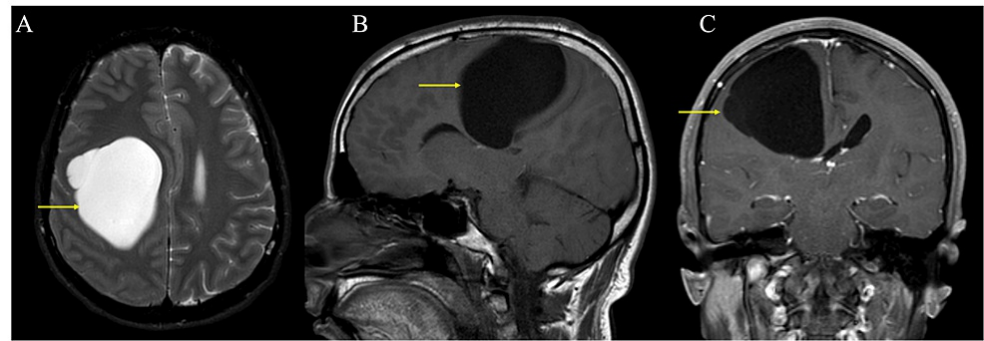
The axial T2 (A), sagittal T1 (B), and coronal CET1 (C) of brain MRI show an intra-axial clear fluid signal intensity cyst within the right posterior frontal lobe (yellow arrows). There is a mass effect of the brain parenchyma and communication with the lateral ventricle. No solid components or abnormal enhancement was noted within the cyst. All the features indicate a diagnosis of a neuroepithelial cyst.

**Figure 8:**
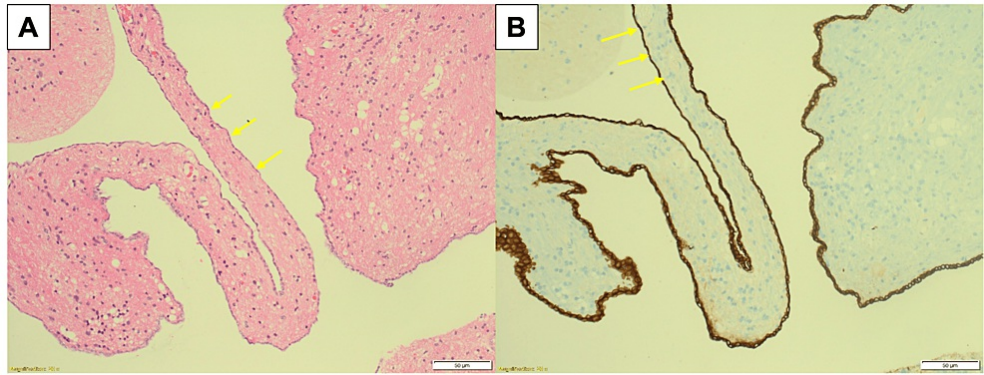
The hematoxylin and eosin-stained section shows a cyst wall lined by simple non-stratified columnar/cuboidal epithelium in (A) (50 μm) (yellow arrows). The EMA immunohistochemistry is positive in the epithelium (B) (50 μm) (yellow arrows). All the features indicate a diagnosis of a neuroepithelial cyst.

STR was done in both cases (100.00%). Both patients had no surgical complications (100.00%) and improved postoperatively (100.00%). No recurrence was noted among the cases within a median of 4.6 (2.2-7) months (Table 3). The radiological and pathological features of one of these cases are shown in Figure 7 and Figure 8.

Rathke’s cleft cyst

Only one case was included in the present study. The patient was a 13-year-old female. On presentation, she had headaches, visual disturbances, vertigo, and hormonal deficiencies. (Table 1). 

On the CT scan, the lesion had a mixed density. On MRI, the lesion was hyperintense on T1 and hypointense on T2 images. Other MRI features included blooming and solid enhancement. The mean lesion volume was 155 mm (Table 2). 

STR has been achieved through surgical management. The patient did not have any complications and her status improved postoperatively. In a follow-up of 18 months, she did not show any recurrence (Table 3).

Xanthogranuloma

Only one case was included in the present study. The patient was a 28-year-old female. She had headaches, nausea/vomiting, and visual on presentation (Table 1). 

On the CT scan, the lesion was hypodense. On MRI, the lesion was hyperintense on both T1 and T2. Other MRI features included solid enhancement. The mean lesion volume was 43 mm (Table 2).

**Figure 9:**
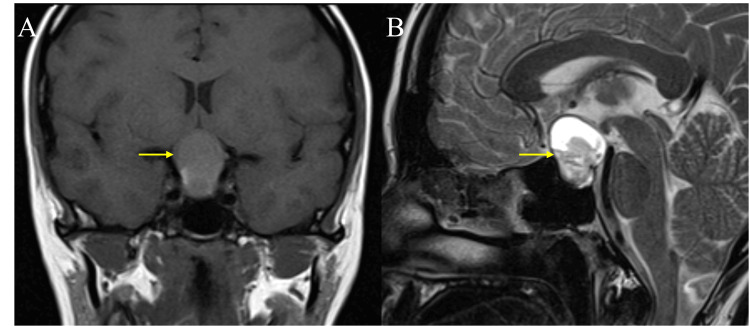
The MRI of the sella turcica shows a well-demarcated solid and cystic sellar mass with suprasellar extension and mass effect on the chiasm and third ventricle. The solid component has a heterogenous appearance on T2 (A) (yellow arrow) and a slightly hyperintense signal on T1 (B) (yellow arrow). All the features indicate a diagnosis of a xanthogranuloma.

**Figure 10:**
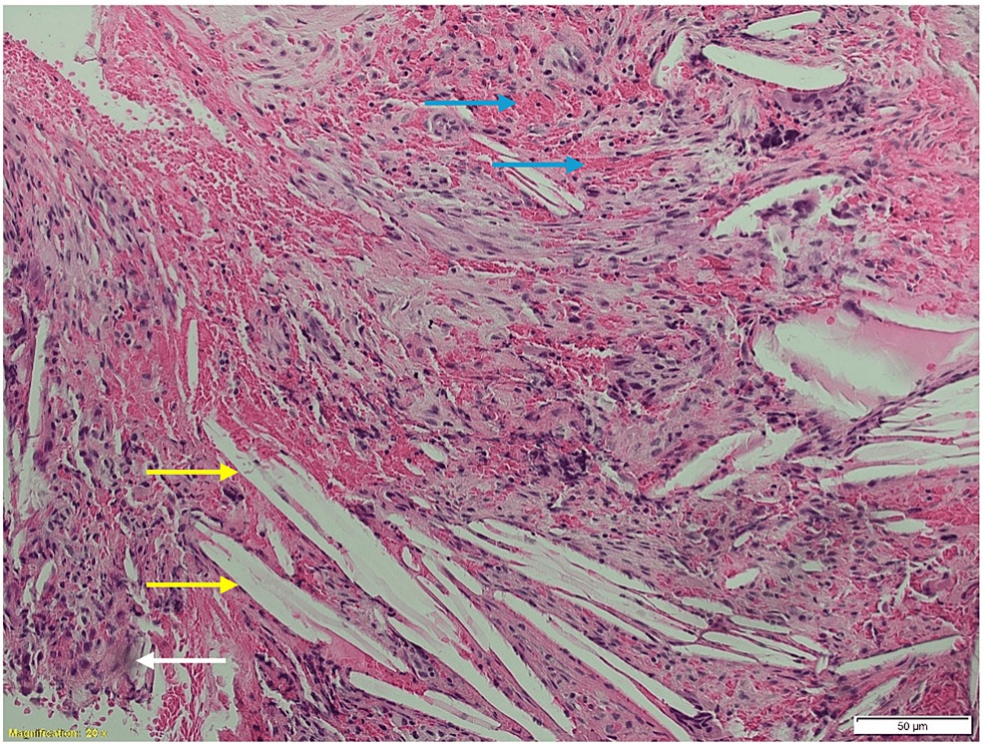
The hematoxylin and eosin-stained section shows cholesterol clefts (yellow arrows), macrophages, chronic inflammatory cells, necrotic debris (blue arrows), and hemosiderin deposits (50 μm) (white arrow). All the features indicate a diagnosis of a xanthogranuloma.

STR has been achieved through surgical management. The patient did not have any complications and her status improved postoperatively. At a follow-up of 19 months, the patient did not show any recurrence (Table 3). The radiological and pathological features of one of these cases are shown in Figure 9 and Figure 10.

Dermoid cyst

Only one case was included in this study. The patient was a 23-year-old male. He had headaches, ataxia, vertigo, and tinnitus/decreased hearing on presentation (Table 1). 

In the CT scan, the lesion was hypodense. No MRI details were available for this case. The mean lesion volume was 80 mm (Table 2).

GTR has been done in this case. The patient developed new-onset seizures postoperatively. However, his preoperative symptoms improved. At a follow-up of 16.33 months, he had no recurrence (Table 3).

## Discussion

Colloid cysts

Colloid cysts are rare benign cystic lesions that are usually found in the anterior third ventricle, and they have the potential to cause hydrocephalus by blocking the foramen of Monro [5]. They have an estimated annual incidence of 3.2 per one million persons [6]. Symptoms tend to develop in the third to seventh decade of life [7]. The median age in our patients was 40 (33-53). The most common symptoms in our patients were headaches, nausea, vomiting, and gait disturbances. This finding is consistent with the literature [8].

Colloid cysts appear hyperdense on non-contrast CT [9]. In MRI, they appear hyperintense in the T1 sequence and isointense or hypointense in T2 [10]. Of our patients who underwent a CT scan, only four (66.66%) had cysts that appeared hyperdense. Six patients (60.6%) had cysts that were hyperintense on T1 and T2 MRI.

Symptomatic cysts are managed by surgical resection through an endoscopic approach or open craniotomy, and a ventricular shunt may be inserted in cases of hydrocephalus [11]. GTR was achieved in only seven (63.63%) of our patients. The prognosis of colloid cysts after resection is generally favorable [8]. Nine of our patients (81.81%) experienced a significant improvement in their symptoms. Postoperative mortality is rare. Of the 11 patients, only one died postoperatively. Postoperative complications included meningitis, memory deficits, focal neurological deficits, cognitive impairment, and seizures [12]. Three (27.27%) in our study experienced postoperative seizures. The recurrence rates have been reported to be 21.2% and 6.7% for endoscopic and microsurgical approaches, respectively [11]. In regard to differential diagnoses of colloid cysts, there are usually none due to their unique radiological appearance and location. This fact makes their preoperative diagnosis accurate in most of the cases [11]. 

Epidermoid cysts

Epidermoid cysts are rare congenital ectodermal inclusion masses that represent 0.2% to 1.8% of all primary brain tumors [13]. When found intracranially, most are in the cerebellopontine angle, parasellar region, or fourth ventricle [14]. Only one (20%) of the patients included in our series, whose MRIs were available, had a supratentorial mass. Most patients present with symptoms between the second and fourth decade of life [15]. The median age of our patients was 26.5 (11.75-41) years. Symptoms resulting from compression of nervous structures by the tumor or hydrocephalus include headache, cranial nerve palsies, gait disturbance, and recurrent aseptic meningitis [16]. The most prevalent presenting symptoms in our patients were headaches, ataxia, and focal neurological deficits.

They appear hypodense on CT and hypointense on T1 and T2 MRI [17]. DWI MRI can be used to differentiate them from arachnoid cysts by showing their solid content [18]. ECs were restricted on DWI MRI in only four (80%) of our patients whose MRIs were available.

Epidermoid cysts are treated by surgical resection, where GTR is preferable but not always possible. In our series, GTR was achieved in only 3 (50%). Incomplete resection has been associated with recurrence [19]. Postoperative complications include facial hypoesthesia, visual deficits, and aseptic meningitis resulting from leakage of cyst content [14]. Halve the patients had permanent neurological deficits postoperatively. Recurrence was seen in two (33.33%) of our patients in a median follow-up period of 30.1 (8.7-49.3) months.

Adamantinomatous craniopharyngiomas

Adamantinomatous craniopharyngioma is a benign epithelial tumor that arises along the craniopharyngeal duct. It has an estimated incidence of 0.19 per 100,000 persons in the United States [20]. They have a bimodal age distribution occurring in children aged five to 14 years and adults between the sixth and eighth decades [21]. All our patients were adults, with a median age of 35 (23-44.5) years. Symptoms include visual field deficits, headache, and endocrine abnormalities [22]. The most common presenting symptoms in our series were headache and visual deficits, while only two (40%) had hormonal deficiency at presentation. 

On CT, they exhibit mixed densities with both cystic and enhancing solid components. Similarly, on MRI, they are of mixed intensity and are enhanced with gadolinium [22]. Calcification on CT is a characteristic that differentiates adamantinomatous from papillary craniopharyngiomas [23]. in our patient, who underwent a CT scan, the lesion was hypodense and had calcification. Only two (50%) of our patients who underwent MRI exhibited mixed-intensity lesions on both T1 and T2 sequences. Peripheral and solid enhancements were observed in all included MRI scans.

Adamantinomatous craniopharyngiomas are managed using GTR, with or without radiotherapy. GTR was achieved in only three (60%) of our patients, and one patient received adjuvant radiotherapy. Postoperative complications included visual deficits, cognitive impairment, and endocrine abnormalities [24]. Hormonal deficiency was the most common postoperative complication in our patients (four (80%)). The recurrence rate was found in one study to be 24% in a mean follow-up of 7.5 years [25]. In our patients, only one had a recurrence during a median follow-up of 42.7 (14.63-45.58) months.

Neuroepithelial cysts

Neuroepithelial cysts are benign congenital masses consisting of sequestered embryonic neural tube remnants that comprise less than 1% of brain tumors [26]. The most common age group at presentation was reportedly the first and third decades of life [27]. Our two patients were aged between eight and 35 years. Frequently encountered symptoms include headaches, focal neurological deficits, and seizures [28]. Seizures, visual impairment, focal neurological deficits, and episodic fainting were the symptoms observed in our patients.

Neuroepithelial cysts appear hypodense on CT, hypointense on T1 MRI, and hyperintense on T2 MRI [27]. In our series, both patients had cysts that were hypointense on T1 and hyperintense on T2. Both cases were supratentorial. 

Open or endoscopic surgical management is the mainstay of treatment for neuroepithelial cysts. Postoperative complications include focal neurological deficits, seizures, and parenchymal hemorrhage [29]. The recurrence rate of neuroepithelial cysts after surgery is not well established. However, this value is low [28]. STR was performed in both patients. Symptomatic improvement was achieved in both patients postoperatively with no postsurgical complications. No recurrence was observed in a median follow-up of 4.6 (2.2-7) months. 

Rathke’s cleft cyst

Rathke's cleft cysts are benign masses arising from remnants of Rathke's pouch. The exact incidence of symptomatic lesions is not known; however, they were found in 11.3% of the autopsies [30]. Their incidence peaks between 30 and 50 years, and they occur more often in females [31]. Common presenting symptoms include headaches, visual deficits, and endocrine abnormalities. Our patient was a 13-year-old girl who presented with a headache, visual disturbance, vertigo, and hormonal deficiencies.

Rathke's cleft cysts appear hypodense on CT, hypointense on T1 MRI, and hyperintense on T2 although the appearance may vary depending on the cyst’s content [32]. They typically do not enhance. In our patient, there was mixed density on CT. On MRI, it was hyperintense on T1 and hypointense on T2. In addition, the cyst exhibited a solid enhancement.

Symptomatic Rathke's cleft cysts are treated surgically, and most patients experience symptomatic improvement after surgery [33]. Endocrine abnormality and cerebrospinal fluid leak are the most common postoperative complications. Reported recurrence rates are variable and can be as high as 48% over a period of five years [34]. Our patient was treated with STR. The patient’s condition improved postoperatively, and no postoperative complications were noted. No recurrence was observed after 18 months.

Xanthogranuloma

A xanthogranuloma is a non-neoplastic, typically sellar mass that results from a chronic inflammatory granulomatous reaction to enclosed cholesterol due to hemorrhage, infarction, inflammation, or necrosis [35,36]. The incidence rate of xanthogranulomas is not well known. In one study, out of 1367 abnormalities identified on MRI, only one (0.07%) was positively identified as xanthogranuloma [37]. The mean age at diagnosis was reported to be approximately 34.7 years [38]. The most common presenting symptoms are headaches, visual deficits, and pituitary hormonal deficiency. Our patient was a 28-year-old female who presented with headaches, nausea, vomiting, and visual deficits.

Xanthogranulomas appear cystic and isodense on CT. On MRI, they are typically hypointense on T1 and hyperintense on T2, although their intensity may vary [39]. Our patient’s CT revealed a hypodense cystic mass. On MRI, the lesion was hyperintense on both T1 and T2, with solid enhancement.

Surgical resection is the mainstay treatment for symptomatic xanthogranulomas, and GTR is often achievable. Radiotherapy may be added if GTR has not been achieved. Common postoperative complications include visual and pituitary hormone deficits. Postsurgical recurrence is a rare occurrence [36,38]. Our patient was treated by STR with no postoperative complications. Her symptoms improved after surgery, and no recurrence was seen at 19 months of follow-up.

Dermoid cyst

Intracranial dermoid cysts are benign lesions that arise from ectopic cellular remnants of the neural tube. They are most commonly found in the posterior fossa and account for 0.1-0.7% of intracranial tumors [40]. A variety of symptoms may result from the mass effect of DCs. In one report, the most common symptoms associated with ruptured dermoid cysts were headaches, seizures, focal neurological deficits, and aseptic meningitis [41]. Symptoms tend to develop between the third and fifth decades of life [42]. Our patient was a 23-year-old male who presented with headaches, ataxia, vertigo, hearing impairment, and tinnitus.

Dermoid cysts appear hypodense in CT [42]. In MRI, they are typically hyperintense on T1-weighted images and hyperintense on T2 images [43]. DWI restriction is rare in dermoid cysts [42]. Our patient’s CT scan showed a hypodense lesion.

Symptomatic dermoid cysts are managed with surgical resection [44]. Postsurgical recurrence is a rare occurrence [42]. Postoperative development of seizures has been previously described [43]. In our patient, GTR was achieved, and his preoperative symptoms improved. The patient developed new-onset seizures after surgery. No recurrence was observed in a follow-up of 16.33 months.

Limitations

This study utilized data from a territorial medical center that treats a significant proportion of IC cases; however, it is essential to recognize its various limitations. The small sample size of this study, including only seven types of ICs, poses a challenge. Moreover, the cases were managed by different neurosurgeons, which may have influenced the outcomes and complication rates. Nevertheless, we hope that we can contribute to the literature on the topic of ICs by providing a comprehensive paper covering the clinicopathological features, radiological features, surgical outcomes and complications, and prognosis of the seven different types of ICs. Further studies with larger sample sizes or highlighting other types of ICs are warranted to provide more insights into this topic.

## Conclusions

ICs are infrequently encountered benign lesions of multiple underlying histopathological origins. This study included and discussed 27 ICs from seven different histopathological origins. Each tumor type exhibited a different pattern in terms of clinicopathological features. Furthermore, based on the type of ICs, specific radiological findings on brain CT and MRI were noted in most cases. Among all cases, surgical management yielded high rates of improvement in preoperative symptoms with low rates of mortality and recurrence; however, surgical complication rates were considerably high.

Recognizing the specific radiological features of each type of IC is an important step in the formulation of a preoperative differential diagnosis. Special medical and surgical considerations based on the type of IC when encountering such cases are a cornerstone of ensuring the best possible outcomes.
